# Intrapreneurial Self-Capital: A Primary Preventive Resource for Twenty-First Century Entrepreneurial Contexts

**DOI:** 10.3389/fpsyg.2019.01060

**Published:** 2019-05-08

**Authors:** Annamaria Di Fabio, Mirko Duradoni

**Affiliations:** ^1^ Department of Education, Languages, Intercultures, Literatures and Psychology (Psychology Section), University of Florence, Florence, Italy; ^2^ Department of Information Engineering, University of Florence, Florence, Italy

**Keywords:** intrapreneurial self-capital, entrepreneurial context, primary prevention, employability, career decision making, innovative behavior, well-being

## Abstract

This article discusses the role of intrapreneurial self-capital (ISC) as a possible primary preventive resource to effectively deal with the complexity of the current entrepreneurial environment. The article deepens both the similarities and differences between ISC and psychological capital and thus proceeds to present the most recent empirical evidence that connects ISC to (1) employability and career decision making, (2) innovative behavior, and (3) well-being. The possibilities for further research and interventions are additionally discussed.

## Introduction

Due to globalization and rapid economic changes, the current labor market is becoming a more unpredictable and challenging environment (Savickas, 2011; Blustein et al., 2018). In the framework defined by Industry 4.0 (Palazzeschi et al., 2018) and by the newborn Industry 5.0 (Özdemir and Hekim, 2018), the psychological aspects of human capital (Luthans et al., 2006) are fundamental for competing in the global market in terms of healthy business (De Smet et al., 2007; Raya and Panneerselvam, 2013; Grawitch and Ballard, 2015; Di Fabio, 2017a) as well as for achieving twenty-first-century sustainability challenges (United Nations, 2018). Modern workers must engage in continuous learning processes, develop flexibility, and create their own opportunities within and outside their organizations (i.e., intrapreneurship and entrepreneurship, respectively) in order to successfully adapt to global economic and technological changes (Standing, 2014; Di Fabio and Gori, 2016b). Since coping with change is often very demanding for certain individuals (Wanberg and Banas, 2000), primary prevention is crucial for building individuals’ strengths and resources as well as reducing risks (Hage et al., 2007; Kenny and Hage, 2009; Di Fabio and Kenny, 2016b) to preserve workers’ well-being and strengthen healthy businesses (De Smet et al., 2007; Raya and Panneerselvam, 2013; Grawitch and Ballard, 2015; Di Fabio, 2017a). Indeed, individuals who manage to perceive and experience change as opportunities to grow are more likely to respond positively to postmodern society’s demands (Wanberg and Banas, 2000; van den Heuvel et al., 2013). From this framework, the new construct of intrapreneurial self-capital (ISC) has been developed (Di Fabio, 2014). The aim of the article is to offer a review of the most recent empirical evidence that connects ISC to (1) employability and career decision making, (2) innovative behavior, and (3) well-being, also discussing possibilities for further research and interventions in a primary prevention perspective.

## Intrapreneurial Self-Capital Construct

ISC refers to a career and life construct, a measurement scale (Di Fabio, 2014), and an intervention typology (i.e., specific training to build and improve the construct) (Di Fabio and Van Esbroeck, 2016). ISC is defined as the positive self-evaluation of the self-concept characterized by one’s own ability to be committed, to identify significant objectives, to feel in control over life events, to creatively solve problems, to change constraints into resources, to develop one’s own skills, to apply decision-making skills to every aspect of life, and to make decisions carefully, vigilantly, and adaptively (Di Fabio, 2014). Basing on the previous literature concerning intrapreneurship, ISC has been conceived as a higher-order construct (i.e., a core of individual resources) that includes seven first-order constructs: *core self-evaluation*, *hardiness*, *creative self-efficacy*, *resilience*, *goal mastery*, *decisiveness*, and *vigilance*. Table 1 illustrates the definition and reference literature of each first-order construct.

**Table 1:** Definition and reference literature for each ISC first-order construct.

First-order construct	Definition	Reference literature
Core Self-Evaluation	A positive self-concept in terms of self-esteem, self-efficacy, locus of control, and the absence of pessimism.	Judge et al., 2003
Hardiness	Individual’s beliefs about the self, the world, and how one should remain connected with this world using three dimensions: commitment, control, and challenge.	Maddi, 1990; Bartone, 1995
Creative Self-Efficacy	One’s perception of one’s own ability to creatively solve problems, including the perception of one’s own problem-solving skills and ability to generate new ideas.	Tierney and Farmer, 2002
Resilience	The perceived ability to cope with adversity in an adaptive way and use adaptive strategies to deal with discomfort and/or adversity.	Campbell-Sills et al., 2006
Goal Mastery	The continuous development of skills and attainment of the highest possible level of performance.	Midgley et al., 2000
Decisiveness	The perceived ability to make decisions in a timely manner in any life context.	Frost and Shows, 1993
Vigilance	The adaptive decision-making style defined as a careful and adaptive search for relevant information to evaluate options before making a decision.	Mann et al., 1997

*The scale developed to measure the ISC construct has a reliability (i.e., Cronbach’s alpha) of 0.86 and indicates appropriate and adequate dimensionality indices [*χ*^2^/*df* = 1.43; Tucker Lewis index or non-normed fit index (NNFI) = 0.90; comparative fit index (CFI) = 0.90; root mean square error of approximation (RMSEA) = 0.05; standardized root mean square residual (SRMR) = 0.04] (Di Fabio, 2014)*.

The scale developed to measure the ISC construct consists of 28 items measured *via* a 5-point Likert scale (ranging from 1 = strongly disagree to 5 = strongly agree). The ISC scale items were selected starting from a pool of items adapted from existing and relevant scales:

- The Core Self-Evaluation scale (Judge et al., 2003).
- Thee Hardiness Scale (Bartone, 1995).
- The Creative Self-Efficacy (Tierney and Farmer, 2002).
- The Connor-Davidson Resilience Scale (Campbell-Sills et al., 2006).
- The Goal Mastery Orientation Scale of the Patterns for Adaptive Learning Survey (Midgley et al., 2000).
- The Indecisiveness Scale (Frost and Shows, 1993).
- The Melbourne Decision Making Questionnaire (Mann et al., 1997).

The pool was statistically tested and the items that best fitted each ISC’s dimension were chosen to compose the final version of the ISC measurement. Below are presented some item examples coming from the final version of the ISC scale: *“Sometimes when I fail, I feel worthless”* (Core Self-Evaluation), *“Planning in advance can help avoid most future problems”* (Hardiness), *“I’m able to solve problems creatively”* (Creative Self-Efficacy), *“I’m able to achieve objectives despite obstacles”* (Resilience), *“One of my goals in training is to learn as much as I can”* (Goal Mastery), *“It’s simple for me to decide”* (Decisiveness), *“When I must to take a decision, I like to stop and consider all possible options”* (Vigilance).

## Article Methodology

We adopted an adapted version of the systematic qualitative review approach (Higgins and Green, 2011) to select which sources involving ISC are discussed in our article. First, we asked to academic information specialists to search for ISC scientific studies encompassing international books, articles, reference works, and conference papers. The specialists conducted their research using EBSCOhost platform and consulting the databases of PsycInfo, PsycArticles, PubMed, Science Direct, Sociological Abstracts, and Academic Search complete. The authors also contributed to the search undergoing a simultaneous check on Google and Google Scholar to improve the chances of identifying the widest range of sources possible. Finally, the authors also inserted in their list all the scientific works that cited the article in which ISC construct has been presented for the first time (Di Fabio, 2014) using Web of Science, Scopus, and Google Scholar tracking system. The sources coming from academic information specialists and authors have been merged in a single dataset and the duplicate sources were removed. At this stage, the sources’ appropriateness were examined and full-text required. For our purposes, we decide to include only peer-reviewed sources in which ISC was discuss or empirically tested along with constructs and measures meaningfully related to work and entrepreneurial research field (e.g., employability, innovation, and well-being). We included only sources written in Italian or English and published between 2014 and January 2019.

## Intrapreneurial Self-Capital and Psychological Capital

Albeit similar and partially overlapping, psychological capital (Luthans et al., 2006) and ISC are two different constructs. ISC refers to a personal wealth in terms of characteristics, abilities, attitudes, and specific skills. This core of resources forms individuals’ selves and promotes an effective management of personal and professional life (Di Fabio and Van Esbroeck, 2016). Psychological capital (PsyCap) is instead an individual’s psychological state characterized by positive psychological resources of efficacy, hope, optimism, and resilience that contribute to the development of the self—including not only who one actually is but also who one may become in the future. Both constructs do share some aspects; for instance, the common features between PsyCap and ISC are the individual’s confidence in his or her own ability to complete an assigned task as well as his or her openness to the future. Moreover, the perception of having enough motivation and energy to optimally plan and realize projects is another shared aspect between ISC and PsyCap. However, it is possible to distinguish between ISC and PsyCap on a theoretical basis, as ISC includes aspects not represented in PsyCap: (1) the careful and adaptive research of the different information available for decision making; (2) the exploration of diversified situations and contexts as opportunities for growth and continuous learning; and (3) the identification of medium- and long-term objectives (Bucci and Di Fabio, 2017). Meanwhile, PsyCap is distinguished by the possibilities: (4) in the present, to experience future paths and outcomes by means of optimistic prefiguration; and (5) to internalize positive elements of the present experiences to nurture a positive vision useful for alternative action strategies in response to contextual stimuli (Bucci and Di Fabio, 2017).

Empirically, the two constructs indicate a strong and positive relationship among both university students and workers: Pearson’s *r* ranged from 0.43 to 0.59 among university students and 0.44to 0.60 among workers (Bucci and Di Fabio, 2017). Nevertheless, the magnitude of the relationship indicates that, despite ISC and PsyCap belonging to the positive preventive perspective framework (Hage et al., 2007; Kenny and Hage, 2009; Di Fabio and Kenny, 2016a,b; Di Fabio, 2017a), the two constructs differ from each other (Bucci and Di Fabio, 2017).

## Intrapreneurial Self-Capital, Employability, and Career Self-Efficacy

Employability is a valuable, individual resource on which individuals can rely to face the current world of work (Di Fabio and Kenny, 2015; Blustein et al., 2018). Several definitions of employability have been produced by employability scholars (Hillage et al., 1998; Fugate et al., 2004; van Dam, 2004; Van Der Heijde and Van Der Heijden, 2006; Rothwell and Arnold, 2007; Fugate and Kinicki, 2008; De Cuyper and De Witte, 2011).

More recently, the concept of sustainable employability has been introduced (van der Klink et al., 2016). Sustainable employability emphasizes the role of the work context in providing workers with new skills and capabilities throughout their working lives. In any case, to increase their employability and thus positively adapt to the labor market, prospective workers should improve their individual resources and qualifications (Di Fabio, 2017a). For this reason, ISC has been studied in relation to employability because, on a theoretical basis, ISC may be considered a possible driver to enhance individuals’ employability. Table 2 summarizes the results originating from correlational studies that account for ISC and employability.

**Table 2:** Correlations between ISC and employability.

	Di Fabio (2014)		Di Fabio et al. (2019)	
	Measured with	Pearson’s *r*	Measured with	Pearson’s *r*
Employability	Self-Perceived Employability (Rothwell et al., 2008)	0.36	Dispositional Measure of Employability (Fugate and Kinicki, 2008)	0.63

*This table refers to ISC relationships with two different measures of employability, one developed and administered to students (left) and the other one dedicated to adult workers (right). The first measure (left) encompasses students’ believes of their future employability, while the second one (right) investigates five employability’s dimensions of adult workers (i.e., work and career resilience, openness to changes at work, work and career proactivity, career motivation, and work identity)*.

Despite their presenting of different Pearson’s *r* correlation coefficients that may be understood in terms of the relationships among two different measures of employability, a positive relationship between ISC and employability emerged in both studies. The ISC appeared to be related to a greater perception of employability. In other words, individuals who have a higher ISC seem more likely to possess personal resources capable of facilitating the identification and achievement of professional opportunities. The different linear relationship strength between ISC and employability expressed through Pearson’s *r* can be due to the different targets for which the two measures of employability have been built, considering the differences between the two constructs of employability. Indeed, the Self-Perceived Employability scale (Rothwell et al., 2008) is intended to investigate students’ believes related to employability, while the Dispositional Measure of Employability (Fugate and Kinicki, 2008) encompasses work and career resilience, openness to changes at work, work and career proactivity, career motivation, and work identity of adult workers.

Another construct that is crucial in helping individuals develop protective factors capable of decreasing the probability of careers’ undesirable outcomes is career decision-making self-efficacy (CDMSE) (Di Fabio et al., 2013). CDMSE has been defined as the level of confidence individuals have in their ability to successfully perform and accomplish tasks during their career decision-making processes (e.g., gathering occupational information, selecting goals, and making plans for the future) (Betz et al., 1996; Betz and Luzzo, 1996; Paulsen and Betz, 2004). The relationship between CDMSE and ISC has been empirically explored (Table 3) among both university students (Di Fabio, 2014) and workers (Di Fabio et al., 2019).

**Table 3:** Correlations between ISC and CDMSE.

	Di Fabio (2014)		Di Fabio et al. (2019)	
	Measured with	Pearson’s *r*	Measured with	Pearson’s *r*
CDMSE	Career Decision Self-Efficacy Scale Short Form (Betz et al., 1996; Betz and Taylor, 2000)	0.46	Career Decision Self-Efficacy Scale Short Form (Betz et al., 1996; Betz and Taylor, 2000)	0.58

As we may gather from Table 3, the ISC was associated with individuals’ greater perceptions of being capable of making decisions regarding their own careers. In this sense, ISC seems capable of promoting workers’ decision-making self-efficacy using a positive primary preventive perspective.

## Intrapreneurial Self-Capital and Innovative Behavior

The psychological aspects of innovation are particularly important for organizations’ success (García-Goñi et al., 2007; Anderson et al., 2014). Organizations try to respond to the uncertainty of the twenty-first century’s labor market though innovation. Traditionally, the scientific literature conceived innovative work behavior as “the intentional introduction and application within a role, group or organization of ideas, processes, products, or procedures, new to the relevant unit of adoption, designed to significantly benefit the individual, the group, the organization, or wider society” (West and Farr, 1990). Intrapreneurs frequently generate and implement new ideas within their organizations even in unfavorable situations (e.g., when facing organizational change and/or conflict) (Pinchot and Pellman, 1999). In this sense, ISC appears to be strongly related to innovative behavior because it includes individuals’ capabilities of coping with work-related issues through innovative solutions (Di Fabio et al., 2017). Recently, the relationship between ISC and innovative behavior has been empirically tested (Duradoni and Di Fabio, 2019). ISC exhibited a positive linear relationship with innovative behavior (Pearson’s *r* = 0.54) measured through the Innovative Behavior Inventory (Lukes and Stephan, 2016). Moreover, higher ISC scores appeared to be related to an increased level of individuals’ abilities to implement innovation (Pearson’s *r* = 0.32). Overall, ISC seems reasonably capable of increasing workers’ innovative behaviors.

## Intrapreneurial Self-Capital and Well-Being

Traditionally, the multi-dimensional construct of well-being (Diener, 2003) has been distinguished by positive psychology scholars (Seligman and Csikszentmihalyi, 2000) using two main aspects: hedonic well-being (HWB) and eudaimonic well-being (EWB) (Ryan and Deci, 2001; Ryff and Singer, 2008; Waterman et al., 2010). HWB is characterized by a cognitive evaluation component (i.e., satisfaction with life) (Diener et al., 1985) and an affective evaluation component (i.e., the prevalence of positive emotions over negative emotions) (Watson et al., 1988), whereas EWB is rather defined in terms of optimal functioning and self-realization (e.g., meaning in life) (Vázquez et al., 2006; Ryff and Singer, 2008). Within the EWB framework, flourishing has received growing attention; this aspect includes purpose in life, positive relationships, engagement, competence, self-esteem, optimism, and contribution toward the well-being of others (Diener et al., 2010; Seligman, 2012; Huppert and So, 2013).

The scientific literature suggests that individual and relational resources are crucial for accessing decent work and maintaining well-being when faced with the new and everchanging world of work (Di Fabio and Kenny, 2016a, 2018). Since ISC is defined in terms of a core of individual intrapreneurial resources, its relationships with both HWB and EWB have been empirically explored. Table 4 illustrates a summary of the results from recent empirical studies concerning ISC in regard to EWB and HWB.

**Table 4:** Study results considering ISC in regard to HWB and EWB.

		Correlation with ISC		
		Di Fabio and Gori (2016c)	Di Fabio et al. (2017)	Di Fabio and Kenny (2018)
Hedonic well-being	Life satisfaction	n/a	*r* = 0.44	*r* = 0.60
	Positive affect	n/a	n/a	*r* = 0.61
	Negative affect	n/a	n/a	*r* = −0.50
Eudaimonic well-being	Meaning in life	n/a	n/a	*r* = 0.69
	Flourishing	*r* = 0.68	*r* = 0.52	*r* = 0.70

*n/a, not available (i.e., the measure was not considered in the study)*.

Higher HWB scores on ISC resources were associated with higher levels of positive affect and global life satisfaction as well as lower negative affect. ISC also resulted in being strongly and positively associated with EWB. Therefore, these recent works suggest that an increase and building up of intrapreneurial resources (i.e., ISC) may be useful in helping individuals achieve and maintain well-being when facing the rapid changes related to our post-modern era (Blustein et al., 2018; Özdemir and Hekim, 2018).

## Intrapreneurial Self-Capital as A Core of Prevention Resources

Historically, prevention has been articulated with three components (Caplan, 1964): primary prevention—i.e., avoiding the emergence of a problem before it begins; secondary prevention—i.e., intervening when first symptoms emerge; and tertiary prevention—i.e., reducing the impact of a problem. The contemporary prevention approach (Hage et al., 2007; Kenny and Hage, 2009) is focused on both reducing risks and building strengths among individuals (e.g., promoting individual resources) (Di Fabio and Saklofske, 2014) as well as within organizations (Hage et al., 2007; Di Fabio, 2017b). Nowadays, the development and increase of specific workers’ strengths are priorities meant to avoid detrimental effects on organizations and workers mainly due to the new entrepreneurial context in which we live, which is characterized by continuous changes and a high degree of uncertainty. Entrepreneurs must continuously face and successfully respond to the challenges inherent to our contemporary entrepreneurial environment (Di Fabio and Gori, 2016b; Özdemir and Hekim, 2018; Palazzeschi et al., 2018). Enhancing innovation and adaptation for changing environments (Renko et al., 2015; Di Fabio et al., 2016) is crucial for successful entrepreneurial behaviors. Nevertheless, it is no longer possible to consider entrepreneurship as a feature detached from other aspects that contribute to shape and define concrete success (Di Fabio et al., 2016) in a liquid and accelerating society, such as our current post-modern society (Bauman, 2000; Rosa, 2015). For instance, the High Entrepreneurship, Leadership, and Professionalism model (HELP) presents entrepreneurship, leadership, and professionalism as integrated, fundamental, and necessary aspects to help workers achieve success in the turbulent twenty-first-century labor market (Di Fabio et al., 2016).

It follows that a primary prevention resource such as ISC, which exhibited positive relationships with innovative behavior, employability, and well-being, is crucial in this new entrepreneurial environment of the twenty-first century. ISC, as a core of individual intrapreneurial and preventive resources, appears to be a primary and vital strength for adaptively dealing with changes and thus increasing the likelihood of successful entrepreneurial behaviors as well as concretely contributing to building healthy businesses (De Smet et al., 2007; Raya and Panneerselvam, 2013; Grawitch and Ballard, 2015; Di Fabio, 2017a).

## Conclusion

The primary prevention approach (Kenny and Hage, 2009) and the psychology of sustainability and sustainable development (Di Fabio, 2017a,b; Di Fabio and Rosen, 2018) benefit from constructs that are likely to be affected by interventions. For instance, personality traits are intrinsic psychological features that slightly change over time and are traditionally considered by literature as being stable (Costa and McCrae, 1992). On the contrary, in regard to the psychological aspects on human capital that emerged in studies subject to modifications (Luthans and Youssef-Morgan, 2017), ISC is an increasable resource (McIlveen and Fabio, 2018). Thus, ISC may potentially introduce new and precious psychological resources to more adaptively handle the changes inherent to our century (Di Fabio and Kenny, 2018). In terms of a primary prevention framework, intervention to enhance ISC could allow to reinforce key aspects in relation to the liquid entrepreneurial environment of the twenty-first century in different targets in a lifelong perspective. ISC could also be applied by practitioners in a career counseling and/or coaching context as well as in other applied contexts. The potentially usefulness of the ISC scale for practitioners working in guidance, career counseling, career planning, life construction, human resources, and organizational development and management is related to the following considerations. People who will receive help in strengthening a set of preventive individual resources as an intrapreneurial core could have more opportunities for handling and succeeding in a constantly changing labor market. Furthermore, an intrapreneurial core could be very important to design one’s own future, to share one’s own opportunities, to improve adaptability skills, to reinforce employability and proactivity, and to construct and manage one’s self, identity, and life (Guichard, 2005; Savickas, 2011). Underlining the value of personal intraprenuership as a primary preventive resource, ISC is also a key resource for flexibly, adaptively, and proactively build one’s own personal and professional development path, enhancing their individual potentialities and talents (Kenny and Hage, 2009; Blustein, 2011). On one side ISC provides career counselors as well coaches with a construct, a scale and an intervention able to inspire and detect a core of primary resources for individuals and workers. On the other side, ISC is promising as a mean to prevent potential career problems instead of focusing on remediation. The new construct calls for early intervention to promote intrapreneurship at school, at the college, in the early transitions to the labor market, in career services but also at organizational and community level, in particular considering entrepreneurial contexts. ISC could be suitable in terms of primary prevention perspective for enhancing people’s early entrepreneurial intentions and possible success of their start-ups (Baluku et al., 2016) as well as in terms of primary and secondary prevention during the construction of the entrepreneurial success.

Nevertheless, a large number of the ISC-related studies present in the literature are correlational. Correlational findings do not offer evidence of causality; thus, longitudinal studies are recommended for assessing change over time and determining the degree of ISC’s and ISC intervention’s impact on the four main areas outlined in this perspective article: (1) employability, (2) career decision making, (3) innovative behavior, and (4) well-being.

Future research should consider additionally possible relationships between ISC and other constructs. For instance, workplace relational civility (Di Fabio and Gori, 2016a) may potentially sustain and amplify the impact of ISC interventions (e.g., establishing a work climate more suitable to face change). Moreover, leadership’s (Di Fabio and Peiró, 2018) (e.g., human capital sustainability leadership) influence on workers’ ISC should be explored, and finally, the possibility to intervene on ISC by means of dedicated web-based training must be tested (Luthans et al., 2008).

Overall, although different, both ISC and PsyCap appear useful for affecting workers’ behaviors and well-being (Avey et al., 2011; Youssef-Morgan and Luthans, 2015; Saks and Gruman, 2017). However, it is crucial that individuals remain aware of the differences between the two constructs. For this reason, one of this article’s aims was to both disambiguate the two constructs by underlining the similarities and differences between them (Bucci and Di Fabio, 2017) and provide scholars and practitioners with useful scientific evidence regarding ISC.

ISC contributes to both HWB and EWB (Di Fabio and Kenny, 2018) as well as other aspects of career development, such as career self-efficacy, career decision making, employability (Di Fabio, 2014; Di Fabio et al., 2019), and innovative behavior (Duradoni and Di Fabio, 2019). Since the entrepreneurial environment is inescapably tied to important, individual psychological needs (i.e., identity, meaning, and personal connection) (Blustein, 2013), the uncertainty and instability ingrained in the current world of work may threaten individuals’ well-being in the workplace (Grawitch and Ballard, 2015; Di Fabio, 2017a; Di Fabio and Kenny, 2018; Duradoni et al., 2018; Giorgi et al., 2019). To successfully handle the current entrepreneurial environment, a broad array of psychological resources and new skills are required. In this sense, ISC may potentially and broadly affect entrepreneurial contexts as a primary preventive resource for building a healthy business that allows individuals to positively cope with threats and challenges inherent to the new, ever-evolving work era.

## Author Contributions

AD and MD ideated the structure, analyzed the literature, and wrote the manuscript.

### Conflict of Interest Statement

The authors declare that the research was conducted in the absence of any commercial or financial relationships that could be construed as a potential conflict of interest.
